# The Diagnostic Pitfalls in the Pronator Teres Syndrome—A Case Report

**DOI:** 10.3390/neurolint17100169

**Published:** 2025-10-12

**Authors:** Wiktoria Rałowska-Gmoch, Marcin Hajzyk, Tomasz Matyskieła, Beata Łabuz-Roszak, Edyta Dziadkowiak

**Affiliations:** 1 Department of Neurology, St. Jadwiga Provincial Specialist Hospital, Institute of Medical Sciences, University of Opole, pl. Kopernika 11a, 45-040 Opole, Poland; wiktoria.ralowska-gmoch@uni.opole.pl (W.R.-G.); beata.labuzroszak@uni.opole.pl (B.Ł.-R.); 2 Orthopedic Children Department, Hospital Complex in Chorzów, Truchana 7, 41-500 Chorzów, Poland; marcin.hajzyk@wp.pl; 3 Department of Neurology, Neurosurgery University of Warmia and Mazury, ul. Warszawska 30, 10-082 Olsztyn, Poland; matyskielat@gmail.com; 4 Clinical Department of Neurology, University Centre of Neurology and Neurosurgery, Faculty of Medicine, Wroclaw Medical University, Borowska 213, 50-556 Wroclaw, Poland

**Keywords:** pronator teres syndrome, proximal median neuropathy, surgical treatment

## Abstract

**Background:** Pronator teres syndrome is a rare proximal median neuropathy caused by compression of the median nerve at various points. It is a rare condition, and many times it is mistaken for carpal tunnel syndrome. **Methods:** There are many authors who refer to the pronator syndrome as a compression of the median nerve at several potential sites of en-trapment in the region of the antecubital fossa, more proximal compression at the Liga-ment of Strutters, and more distally, including lacerus fibrosus within the pronator teres muscle and the anterior interosseous nerve. **Results:** The diagnostic difficulties in a patient with severe right forearm pain during elbow flexion and pronation are presented. Routine test results, including MRI of the right elbow joint, nerve conduction study of the brachial plexus and ulnar nerve, and electromyographic study of the muscles of the right upper ex-tremity, were normal. Ultrasonography showed an enlarged pronator teres muscle. **Conclusions:** The patient underwent surgical removal of the lacertus fibrosus. All symptoms resolved.

## 1. Introduction

Pronator teres syndrome (PTS) is a proximal median neuropathy caused by compression of the median nerve at various points within the anatomical structures of the elbow and forearm. It is a rare condition, and many times it is mistaken for carpal tunnel syndrome (CTS). There are many authors who refer to the pronator teres syndrome as a compression of the median nerve at several potential sites of entrapment in the region of the antecubital fossa, more proximal compression at the ligament of Struthers, and more distally, including lacertus fibrosus within the pronator teres muscle [1,2,3,4,5]. Others describe PTS as a compression of the median nerve by only the pronator teres muscle (PT) in the forearm [1].

Among the causes of PTS are repetitive gripping or forearm pronation movements (e.g., prolonged hammering, scooping food, dishwashing, playing tennis). These movements can lead to muscle hypertrophy in the antecubital fossa and result in median nerve damage, especially in individuals with additional fibrous bands [1]. In the scientific literature, PTS has also been described as resulting from local trauma, compression with schwannoma, anticoagulation therapy, and dialysis [1,5].

Clinical symptoms depend on the location and cause of the lesion. Pain and discomfort usually occur at the site of nerve entrapment [1,3,5,6,7,8,9]. In proximal median neuropathy, sensory loss affects the thenar eminence, thumb, index, middle, and lateral ring fingers, while in carpal tunnel syndrome (CTS), the thenar eminence is spared [3,4,5]. Struthers syndrome, a rare compressive neuropathy, should be considered in the differential diagnosis. Here, the median nerve or brachial artery is compressed by the ligament of Struthers and the humeral supracondylar process, causing hand pain, numbness, weakness, or ischemic forearm pain during resisted elbow flexion [8,9,10,11,12,13]. Differentiating pronator teres syndrome (PTS) from CTS, anterior interosseous nerve syndrome (AINS), cervical radiculopathy, and thoracic outlet syndrome is essential. PTS occurs with CTS in 6–11.5% of cases [12]. Lacertus fibrosus syndrome involves median nerve compression below the bicipital aponeurosis, just distal to the elbow. It is associated with repetitive forearm movements and presents mainly as reduced hand strength and endurance [14].

**Figure 1:**
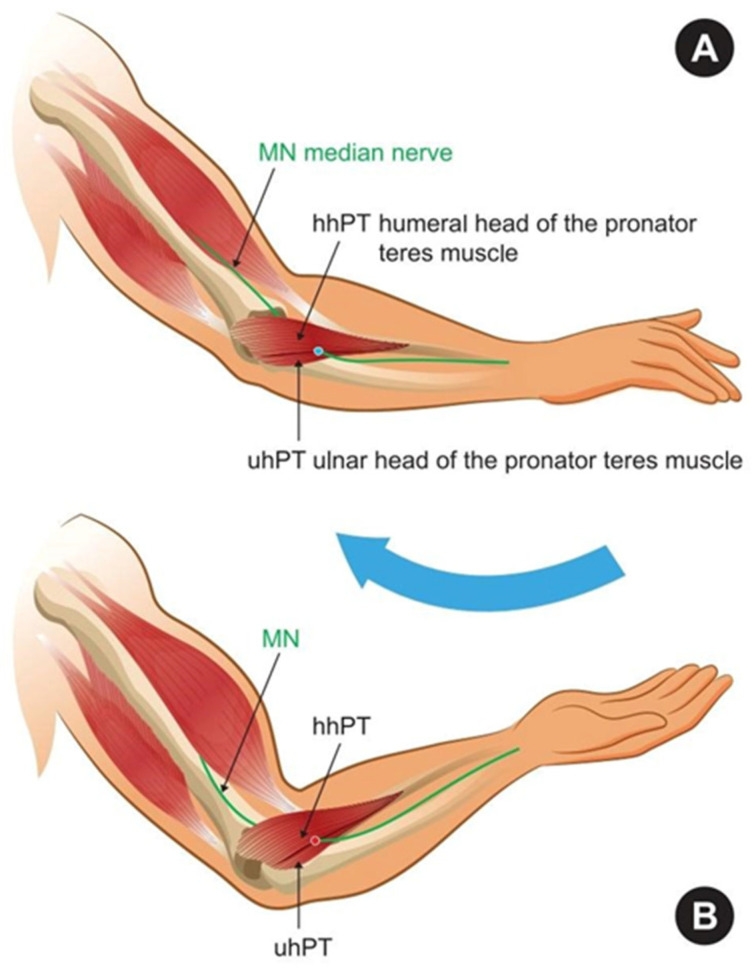
Schematic illustration of the provocative test for pronator teres syndrome: (**A**)—Neutral position without compression of the median nerve; blue dot—absence of symptoms. (**B**)—Forearm pronation results in compression of the median nerve and the onset of symptoms; red dot—elicitation of symptoms.

One of the easiest provocative tests for PTS is: pronator teres resistant pronation or activation of the sublimity bridge of the flexor digitorum superficialis (FDS) by resisted flexion of the long finger when other fingers are held in extension, or activation of the lacertus fibrosus with resisted flexion of the elbow with forearm in supination. One can provoke PTS symptoms by compression of the nerve between the two heads of the pronator teres muscle during resisted forearm pronation in the extended elbow (Figure 1) [2,15,16,17,18,19]. The Tinel’s sign at the proximal forearm, and a positive Phalen test over the pronator teres muscle can be present in 50% of cases [1].

Electrodiagnostic studies are very important to localize the lesion as a result of trauma or compression, and neuromuscular ultrasound is a complementary test. Very often, the routine tests are normal or nonspecific [11,12,13,20,21]. Electromyography (EMG) abnormalities are most commonly observed in the flexor pollicis longus (FPL) and flexor digitorum profundus (FDP) to digits 2 and 3, less frequently in the flexor digitorum superficialis (FDS) and abductor pollicis brevis (APB) [1,13,20,21,22]. EMG should assess median-innervated muscles both proximal and distal to the carpal tunnel to differentiate PTS from CTS, radiculopathy, or AINS. Additionally, at least two non-median C8–T1 and one non-median C6–C8 muscles should be examined to exclude brachial plexopathy or radiculopathy [1,2,8,23].

Ultrasonography has an important role in diagnosing PTS and other upper limb neuropathies. It is a useful tool for the examination of PT and dynamic changes in the morphology of the median nerve. Magnetic resonance imaging may show a hyperintense signal change secondary to denervation edema of the anterior forearm muscles [10,11,13,20,21,24,25].

The goal of pronator teres syndrome treatment is to relieve median nerve compression and restore function. Conservative management—rest, immobilization, physical therapy, and braces—aims to reduce nerve pressure and improve strength, flexibility, and motion. If symptoms persist after 3–6 months, surgical treatment may be considered [12,16,22,25,26,27,28].

Surgical management involves identifying and protecting the medial and lateral antebrachial cutaneous nerves, followed by forearm incision, median nerve exploration, and decompression by releasing the pronator teres and other compressive structures, including the ligament of Struthers, lacertus fibrosus, and the FDS fibrous arch [22,29,30]. All tendinous portions of the pronator teres must be released during a single-stage procedure. Decompression at multiple sites is still considered one operation [1,29,30,31].

The prognosis of pronator syndrome depends on the timing of diagnosis, severity of nerve compression, and effectiveness of treatment interventions. In initial appropriate therapy, the degree of nerve entrapment has an influence on treatment results [20,22,29,30,31,32,33].

The aim of this study was to present a comprehensive clinical overview of a patient diagnosed with pronator teres syndrome, with particular focus on the underlying anatomical factors, diagnostic challenges, and therapeutic management. The authors provide a detailed discussion of the differential diagnosis, highlighting the potential for misdiagnosis with other neuropathies of the upper limb. Accurate identification of the syndrome allowed for the implementation of surgical intervention, which resulted in complete resolution of the patient’s symptoms and significant functional recovery. This case underscores the importance of careful anatomical and clinical evaluation in the management of peripheral nerve entrapment syndromes.

## 2. Case Report

A 34-year-old man was referred to our clinic because of strong pain in the right forearm during flexion of the elbow, pronation movements, pain around the pronator teres palpation, and sensory disturbances involving the entire palm of his right dominant hand. The initial symptoms included mild symptoms in the middle finger. Retrospectively assessed using the 2-point Visual Analog Scale (VAS) for pain. The symptoms persisted for over one year. He was working as a carpenter, and because of the pain, he could not work and avoided activities that required repeated pronosupination movements. His symptoms progressively intensified due to his activities and occupation. He was very healthy, and he did not have any significant medical or surgical history.

The medical examination reveals pain in the forearm region aggravated by resisted pronation of the forearm and flexion of the elbow. Pain intensity was assessed as 9 points on the VAS. The pronator compression test was positive around PT, while the Carpal Tunnel Test, like the Tinel, Phalen test, showed negative. The Adson test was normal.

On physical examination, thenar muscle atrophy was found in the right hand, and the index and middle finger flexion strength was 4/5 Medical Research Council Scale, the thumb abduction strength was 4/5, and the ring and little finger flexion strength was 5/5. Hypoesthesia was found in the median nerve territory. The deep tendon reflexes were normal, and the patient was able to make an “OK sign”.

**Figure 2:**
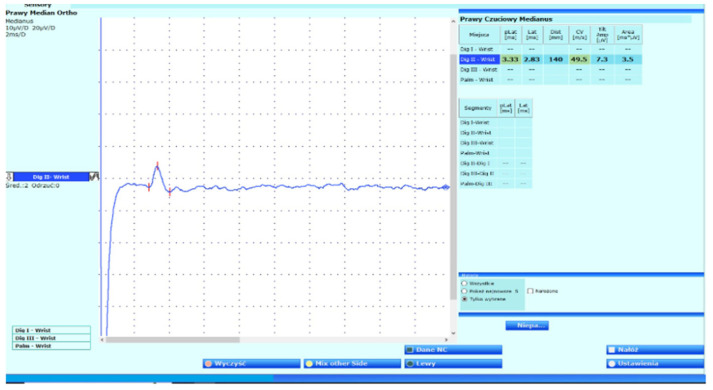
Prolonged latency of the sensory nerve action potential of the median nerve; normal value: 3.20 ms.

Autoimmunological and metabolic tests were normal. Borelioza, Human Immunodeficiency Virus (HIV), Venereal Disease Research Laboratory (VDRL), and Creatine Kinase (CK) tests were normal. Cervical ribs were not visible on X-ray, and Doppler ultrasound of the neck and upper limb vessels revealed no pathology. In the Magnetic Resonance (MR) scan of the right elbow and adjacent muscles, there was no discernible pathology. An MR scan of the neck and cervical spine was normal. The routine nerve conduction study revealed only prolonged sensory median latency (Figure 2). All other nerves of the brachial plexus with ulnar inching were normal; additional tests excluded CTS. Normal conduction parameters were obtained for the anterior interosseous nerve. Nerve conduction studies also did not provide evidence of thoracic outlet syndrome. Extensive EMG of the affected limb was normal.

Despite consultation with several medical professionals, a final diagnosis could not be determined.

He was treated with injections of steroids and physical therapy, but there were no improvements. The patient was treated with anti-inflammatory drugs and Gabapentin 1200 mg/d, Pregabalin 600 mg/d without improvement. Ultrasound-guided hydrodissection around the median nerve with corticosteroid injection also did not reduce the symptoms.

He was also rehabilitated using physiotherapy, kinesiotherapy, therapeutic exercises, and manual techniques that aid in stretching and releasing the compressed nerve. A full rehabilitation protocol was made, but there were no significant improvements.

Muscle ultrasound showed the enlarged pronator teres. The patient underwent the orthopedic consultation and then the removal of the lacertus fibrosus. The approach was a most uncommon skin incision, but it was still a modification of Henry‘s surgical instruction. The approach was S-shaped and approximately 10 cm. Cutaneous nerve branches of the lateral brachial and medial antebrachial cutaneous nerve were identified and atraumatically mobilized.

**Figure 3:**
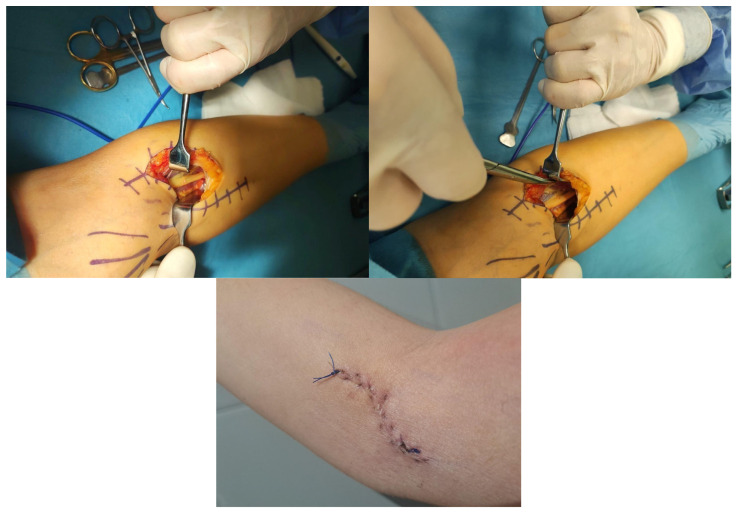
Surgical incision-“lazy S “volar incision for proximal median nerve decompression is made 3 cm distal to the medial epicondyle over the pronator muscle.

The easiest way to find the medial nerve was to look for the proximal part in the elbow flexion crease. The lacertus fibrosus was cut. After that step, the existence of Struher’s ligament and supracondylar process could be seen. The radial artery lay radial to the nerve and had to be protected throughout the procedure. The medial nerve was adherent to the pronator teres muscle. The muscle mass was protected, and the dissection proceeded to the distal part of the superficial arcade. The tendinous portion of the pronator teres was released. It was critical for all tendinous portions of the pronator teres that potentially compressed the nerve to be released in one step (Figure 3).

Two weeks after surgery, the patient was getting better, and the tingling sensations were much improved. One month later, the Tinel sign was negative. Motor power of the first and third fingers had improved to 5 in the Medical Research Scale. His complaints significantly lessened, as depicted by VAS. Shortly thereafter, he came back to his occupation and lived a normal life free of pain.

At the neurological follow-up visit six months after surgical treatment, clinical improvement was maintained, and the patient reported no recurrence of symptoms. He remained professionally active.

## 3. Summary

Pronator teres syndrome is a rare condition that is more common in women and people who perform repetitive forearm and wrist movements. Usually, it is difficult to diagnose because it can mimic other, more common conditions, such as CTS. Clinical diagnosis is based on diagnostic and imaging studies. This case report showed us to never give up, even when the diagnostic tests are nearly normal, but clinical features are dominant during patient examination. Physical therapy is a mainstay treatment for PTSD, as well as anti-inflammatory drug therapy and corticosteroid injections, which should be required for at least 6 weeks before any surgical interventions.

The management of pronator teres syndrome is challenging and requires consideration of various therapeutic options. The literature increasingly emphasizes the importance of ultrasound-guided hydrodissection when conservative measures fail to improve symptoms [16,34,35,36]. Shojaie et al. described their experience in treating pronator teres syndrome in a healthy young badminton player using ultrasound-guided median nerve hydrodissection. Following the initial session, the patient reported a 50% reduction in VAS score and improved capacity to lift weights. A second session was administered four weeks later. At the three-month follow-up, the VAS score had declined to 1/10, and ultrasonographic assessment demonstrated a normal median nerve with a negative sonographic Tinel sign, and the patient returned to professional badminton pain-free [34].

Particular attention should be given to delayed onset muscle soreness (DOMS). DOMS is well recognized after unaccustomed or resisted exercises of large muscles, but is often overlooked in the intrinsic and extrinsic muscles of the hand. DOMS reflects exercise-induced muscle damage (EIMD). According to Tedeschi, recognizing this phenomenon can improve exercise prescription, enhance recovery, and prevent setbacks in rehabilitation [37]. Also, Szajkowski et al. emphasize that after DOMS, foam rolling and percussive massage accelerate the restoration of muscle tone, decrease stiffness, and improve elasticity relative to passive rest, but offer no extra benefit for pain reduction [38]. However, the current literature does not support a clear advantage of foam rolling or stick massage in improving indirect markers of muscle damage (soreness, range of motion, swelling, and maximal voluntary isometric contraction) compared to no intervention in healthy individuals. Methodological heterogeneity and the limited number of high-quality studies make it difficult to draw definitive conclusions [37,38,39,40].

## 4. Conclusions

Pronator teres syndrome describes the signs and symptoms that result from compression of the median nerve by the pronator teres muscle in the forearm. The clinical presentation is different from its more common distal counterpart, carpal tunnel syndrome. Therefore, it is very important to correctly diagnose the pronator teres syndrome and provide appropriate treatment.

## Data Availability

Informed consent was obtained from all subjects involved in the study. The diagnostic procedures were conducted in accordance with the principles outlined in the Helsinki Declaration (1975, revised in 2013). Resolution No. KB 314/2024, issued on 18 April 2024, by the Bioethics Committee of Wrocław Medical University.

## Abbreviations

AINS	Anterior Interosseous Nerve Syndrome
APB	Abductor Pollicis Brevis
CTS	Carpal Tunnel Syndrome
DOMS	Delayed Onset Muscle Soreness
EIMD	Exercise-Induced Muscle Damage
EMG	Electromyography
FDS	Flexor Digitorum Superficialis
FDP	Flexor Digitorum Profundus
FPL	Flexor Pollicis Longus
PT	Pronator Teres Muscle
PTS	Pronator Teres Syndrome
VAS	Visual Analog Scale
